# A case study of critical points for the entry and spread of *Salmonella* Infantis in a broiler farm

**DOI:** 10.1186/s13620-025-00329-9

**Published:** 2026-01-07

**Authors:** Bojan Papić, Jasna Mićunović, Brigita Slavec, Neva Šemrov, Olga Zorman Rojs, Jana Avberšek

**Affiliations:** 1 University of Ljubljana, Veterinary Faculty, Institute of Microbiology and Parasitology, Gerbičeva 60, Ljubljana, 1000 Slovenia; 2 University of Ljubljana, Veterinary Faculty, Institute of Poultry, Birds, Small Mammals and Reptiles, Gerbičeva 60, Ljubljana, 1000 Slovenia; 3 VET.AM.JATA d.o.o., Slomškova ul. 30, Domžale, 1230 Slovenia

**Keywords:** *Salmonella* Infantis, Critical points, Whole-genome sequencing, Broiler farm, Transmission

## Abstract

*Salmonella* Infantis is a zoonotic pathogen that frequently persists in broiler production systems, posing a risk to food safety and animal health. Understanding its entry and spread in these systems can inform control measures and reduce contamination.

A longitudinal field study (2021–2022) was conducted on an eight-house broiler farm managed by three farmers to identify critical contamination points for *S*. Infantis. In 2021, 49 out of 536 samples (9.1%) were positive for *S*. Infantis, with the highest contamination rates found in anterooms (11.7%), the farm exterior (9.1%) and in the production area (7.5%). Only two out of 196 samples were positive after the implementation of cleaning and disinfection (C&D) measures. After destocking, 5.9% of samples tested positive for *S*. Infantis. The critical points with the highest contamination rate were litter, ventilation systems and nipple cups in the production area, floor and equipment in the anteroom and farm exterior. When the farm was revisited in 2022, *S*. Infantis was detected in four houses after the fattening cycle was completed, although there was no contamination prior to flock arrival. Whole-genome sequencing of 69 isolates showed that all isolates harbored the pESI plasmid and belonged to ST32. Clonal *S*. Infantis isolates mostly originated from houses managed by the same farmer, with occasional cross-contamination between houses managed by different farmers. Although persistent strains were frequently observed, the introduction of a new strain into previously negative houses was also observed.

This study highlights that strict and consistent adherence to C&D protocols is critical for effective control of *S*. Infantis spread within and between flocks. Persistent contamination in certain areas highlights the need for targeted interventions at identified critical points.

**Supplementary Information:**

The online version contains supplementary material available at 10.1186/s13620-025-00329-9.

## Background

Salmonellosis is the second most common gastrointestinal infection in EU/EEA countries and an important cause of foodborne outbreaks [1]. In EU/EEA countries, the incidence of salmonellosis was 18.0/100,000 in 2023 [1]. *Salmonella enterica* serovar Infantis is the fourth most common serovar associated with human salmonellosis in EU/EEA countries [1]. It is also the most prevalent serotype in broilers and the third most reported serovar in laying hens and fattening turkeys in EU/EEC countries [1, 2].

*S.* Infantis is a poultry-adapted *Salmonella* serovar with a high ability to spread and persist in broiler farms, indicating its exceptional environmental persistence [3, 4]. Currently, *S.* Infantis in broiler farms is mainly controlled by biosecurity measures, cleaning and disinfection (C&D) protocols and good management practices, which are not always sufficient to completely and permanently eradicate *S.* Infantis from persistently infected broiler farms [3, 5, 6]. *S.* Infantis spread in a broiler farm is mostly horizontal (i.e. through environmental contamination), although vertical transmission from hatcheries can also occur [7, 8]. Well-known risk factors for *Salmonella* contamination include the presence of the pathogen in the barn and in the previous flock, house status after C&D, the presence of the pathogen in day-old chicks, contaminated feed and water [9, 10]. *S*. Infantis was also found more frequently in broiler flocks using coccidiostat salinomycin, where flock thinning was carried out or litter quality was poor [8]. Pathogen presence in the barn environment promotes recontamination of broilers with *S*. Infantis, which increases the pathogen load in the barn environment through fecal shedding [11].

In the last decade, *S*. Infantis lineage that harbors a plasmid of emerging *S*. Infantis (pESI) has spread worldwide and has replaced the pESI-free *S*. Infantis population [12, 13]. This megaplasmid encodes genes involved in antimicrobial resistance, biofilm formation and disinfectant tolerance, thereby increasing host fitness. The plasmid also increases host virulence, including its adhesion and invasion in mammalian and avian cells [14–16]. pESI therefore likely promotes the persistence of *S*. Infantis in broiler farms. Recently, MDR-encoding pESI-like plasmids have also been found in certain other *Salmonella* serovars, posing an additional problem for human and animal health [17, 18].

A previous study investigated persistently contaminated broiler farms in the UK found that regular on-farm C&D measures need to be extended to the farm exterior and feeder lines to successfully eradicate *S.* Infantis [5]. Recently, new and holistic approaches combining regular C&D measures with targeted biocontrol agents (bacteriophages), microbiota modulation and/or vaccination have also been increasingly investigated [6, 19–21].

The identification of critical points for the introduction and spread of *S.* Infantis in the broiler industry enables the implementation of effective and targeted control and prevention measures, thus reducing the risk of contamination. Worldwide, whole-genome sequencing (WGS) is becoming the method of choice for pathogen surveillance and enables accurate identification of transmission clusters. To our knowledge, only a few studies have used WGS to identify the persistence and transmission routes of *S*. Infantis within broiler farms [8].

## Case presentation

A longitudinal field study of *S.* Infantis contamination in a broiler farm was conducted to (*i*) identify the critical points for the introduction and spread of *S.* Infantis in a broiler farm and (*ii*) compare the persistence of *S*. Infantis within a barn before and after standard C&D. To this end, a thorough bacteriological examination of the barns and farm surroundings was performed, followed by WGS of the obtained *S*. Infantis isolates.

## Methods

### Study design

A broiler farm with a history of *S*. Infantis was investigated between February 2021 and July 2022. The farm consisted of eight houses maintained by three farmers contracted to the same poultry company. Farmer A maintained houses 1, 2 and 3, farmer B maintained houses 4, 5 and 6, and farmer C maintained houses 7 and 8. All houses were single-story conventional buildings with a capacity of 20,500 broilers and an all-in–all-out system. They had concrete floors and were equipped with either manually or automatically controlled ventilation systems, automatic nipple drinkers with spilling cups and automatic feeders. The birds were reared on wood shavings. All chicken flocks originated from the same hatchery, had the same feed supplier and were slaughtered in the same slaughterhouse. After removal of the flocks, all houses were dry-cleaned, washed, and disinfected. The turnaround time was 2–3 weeks.

The standard C&D procedure included the removal of old bedding (manure) and leftover feed, followed by mechanical cleaning of the floor, walls and equipment, including drinkers and feeders. After dry cleaning, the walls, floor and equipment surfaces were washed with water. In all houses, an alkaline cleaner (Kenosan, CID LINES N.V., Belgium) was applied to the floor, walls, feed and water pipes and removed after 1–2 h by rinsing with water. High-pressure washing was used in houses 7 and 8, whereas the other houses were washed using water supply pressure. The ventilation system was cleaned by blowing out. For the first and final disinfection, Virocid (CID LINES N.V., Belgium) was used in all houses.

### Sampling

**Fig. 1:**
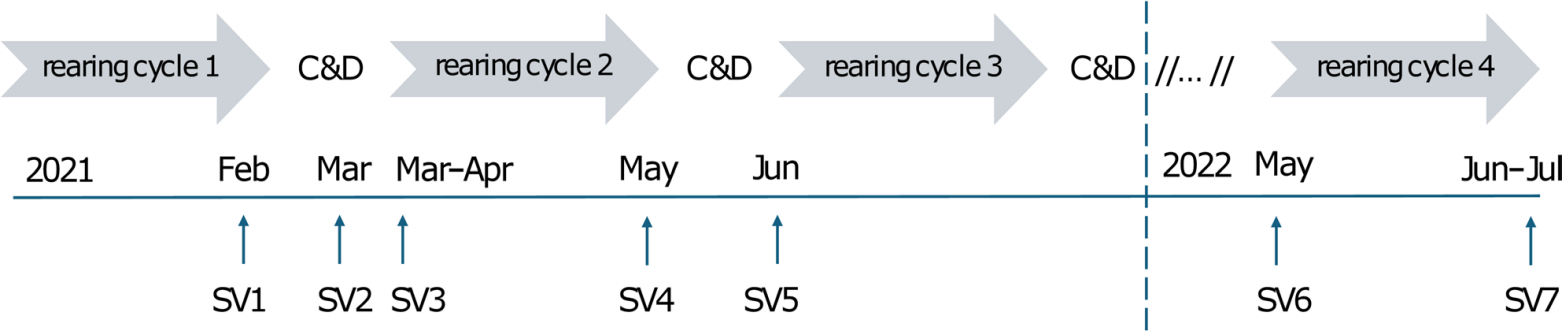
Timeline of the sampling visits (SVs)

In total, seven sampling visits (SVs) were carried out between February 2021 and July 2022 (Fig. 1). The sampling protocol (e.g. number and type of samples) and frequency varied between visits according to housing-in and destocking, which varied between houses and were influenced by the defined critical points in previous SVs. The number and type of samples obtained in each SV is shown in Tables 1 and 2, respectively and per each house in Tables S1 and S2. SV 1 included a large-scale sampling that was done immediately after destocking (i.e. at the completion of rearing cycle 1). Each house was sampled to assess the contamination rate during the rearing cycle. In each house (1–8), samples were collected in the production area, in the anteroom and in the immediate house exterior. Floor/litter samples were collected using a pair of boot swabs. At the same time, samples were collected from the fans, air flaps/ventilation system, feeders, feed hopper/feeding system, nipple cups and other samples (e.g. surface of water pipes and lights). In addition, two water samples were taken at the beginning and at the end of the water line of the nipple system. In the anteroom, samples were taken from the floor, the disinfection bath, from fixed or movable equipment such as water tap and funnel, storage shelves, freezer for cadavers (cadaver chest), wheelbarrow and brooms. The area around each house was also sampled. In front of the house, concrete floors, grass and vehicle-will-deep at the farm entrance were sampled (house surroundings), and samples were collected from silos and cats. Vehicles on the premises (trucks for transporting broilers to the slaughterhouse, trucks for transporting feed and vans for transporting workers helping with destocking) were also sampled. In addition, all three farmers agreed to sample their cars (tires and vehicle interiors), footwear, working clothes as well as their hands and hair. SV 2 (between rearing cycles 1 and 2) took place after washing and cleaning but before disinfection and thus served to assess the efficiency of the cleaning measures. Standard cleaning protocol with proposed modifications described further in the text was used. During the visit, the condition of the poultry houses was assessed together with the farmers. Based on this assessment and the results of the initial sampling, recommendations were provided regarding cleaning and disinfection and other control measures to facilitate the elimination of *S.* Infantis from the barns and farm exterior. During the assessment, residual dirt and dust was found on individual parts of the facilities and on equipment. For cleaning, the importance of soaking to remove residual dirt from surfaces before soaping was emphasized, with particular attention to drinkers and feeders. For washing, the use of high-pressure machines was recommended, as well as the use of an acid descaler for drinkers. The importance of rinsing detergents from surfaces within one hour of exposure was emphasized to remove both the dirt and the detergent itself. Farmers were urged to use protective clothing, footwear and other equipment exclusively within a single poultry house. They were advised to thoroughly clean and disinfect the environment outside the houses by applying lime and to exercise greater control over vehicles entering the farm. Samples (SV 2) were collected from the interior of each house including the floor, air flaps (ventilation system) and nipple cups. SV 3 was conducted on the day of chicken arrival (rearing cycle 2). The sampling protocol in SV 3 was the same as for sampling visit 1, while in SV 4 (after destocking at the end of rearing cycle 2), the sample number and sample types varied depending on the results of the isolation of *S*. Infantis in the previous samplings. Additionally, in house 3, flies found dead in the anteroom were also sampled. SV 5 (before the start of rearing cycle 3) was performed only in house 3, in which *S.* Infantis was detected in the slaughterhouse. The sampling protocol in SV 5 was the same as in SV 1 (except the sampling of the cats was not performed in SV 5). After approximately one year, the farm was revisited in 2022, and samples were taken in all houses at the arrival of chickens (SV 6) and on the day of slaughter of the chickens of the same rearing cycle (SV 7). SVs 6 and 7 had a lower sampling effort and were adapted to the results of the previous samplings since they were aimed to identify potential persistent *S*. Infantis strains. In addition, five neck skin samples per house were collected in the slaughterhouse after the first and second destocking (SVs 1 and 4) and one year later (SV 7).

**Table 1:** *Salmonella* Infantis contamination rate in samples collected during sampling visits 1–5 in 2021. The number of positive samples vs. the total number of samples is provided in the last column.

		Sampling visit					
Area	Sample type	1 (Rearing cycle 1, after destocking)	2 (Between cycles 1 and 2, before disinfection)	3 (Rearing cycle 2, before housing)	4 (Rearing cycle 2, after destocking)	5^*^ (Rearing cycle 3, before housing/ house 3)	No. of positive samples/No. of all samples (%)
**Production area**	Floor/litter	**2/8**	**1/8**	0/8	**1/8**	0/1	**4/33 (12.1)**
	Ventilation system	**4/16**	0/8	0/9	0/2^*^	0/1	**4/36 (11.1)**
	Feeding system	**1/16**	NS	0/15	0/2^*^	0/2	**1/35 (2.9)**
	Nipple cups	**4/8**	0/16	0/16	0/8	0/2	**4/50 (8.0)**
	Water from the water pipe	0/8	NS	0/8	**1/1** ^*^	0/1	**1/18 (5.6)**
	Other	**2/19**	NS	0/16	0/4^*^	0/2	**2/41 (4.9)**
	**Total**	**13/75**	**1/32**	**0/72**	**2/25**	**0/9**	**16/213 (7.5)**
**Anteroom**	Floor	**6/8**	NS	0/8	**2/8**	0/1	**8/25 (32.0)**
	Disinfection bath	**1/8**	NS	0/8	0/1^*^	0/1	**1/18 (5.6)**
	Equipment surfaces	**2/22**	NS	**1/22**	0/3^*^	0/3	**3/50 (6.0)**
	**Cleaning tools**	**1/8**	NS	0/8	0/1^*^	0/1	**1/18 (5.6)**
	**Total**	**10/46**	/	**1/46**	**2/13**	**0/6**	**13/111 (11.7)**
**Farm exterior/** **vehicles**	Silo	0/8	NS	0/7	0/1^*^	0/1	**0/17**
	House surroundings	**4/6**	NS	0/7	0/4^*^	0/3	**4/20 (20.0)**
	Cats	**1/5**	NS	NS	NS	NS	**1/5 (20.0)**
	Farmers	**2/12**	NS	0/12	0/4^*^	0/4	**2/32 (6.25)**
	Farmers’ vehicles	**1/6**	NS	0/8	0/2^*^	0/2	**1/18 (5.6)**
	Trucks/feed, day-old chicks	0/4	NS	0/12	NS	0/2	**0/18**
	Truck/destocking	**2/6**	NS	NS	**1/2** ^*^	NS	**3/8 (37.5)**
	Other	0/3	NS	NS	NS	NS	**0/3**
	**Total**	**10/50**	/	**0/46**	**1/13**	**0/12**	**11/121 (9.1)**
**Slaughterhouse**	Neck skin	**8/40**	NS	NS	**1/40**	NS	**9/80 (11.3)**
	Other	NS	NS	NS	0/11	NS	**0/11**
	**Total**	**8/40**	/	/	**1/51**	/	**9/91 (9.9)**
**No. of positive samples/No. of all samples (%)**		**41/211 (19.4)**	**1/32 (3.1)**	**1/164 (0.6)**	**6/102 (5.9)**	**0/27**	**49/536 (9.1)**

Positive samples are shown in bold. ^*^Sampling visit was performed only in house 3. NS, not sampled

**Table 2:** *Salmonella* Infantis contamination rate in samples collected during sampling visits 6–7 in 2022. The number of positive samples vs. the total number of samples is provided in the last row.

		Sampling visit		
**Area**	Sample type	**6** (Rearing cycle 4, before housing)	**7** (Rearing cycle 4, after destocking)	**No. of positive samples/No. of all samples (%)**
**Production area**	Floor/litter	0/8	**2/8**	**2/16 (12.5)**
	Ventilation system	0/8	0/8	**0/16**
	Nipple cups	0/8	0/8	**0/16**
	**Total**	**0/24**	**2/24**	**2/48 (4.2)**
**Anteroom (total)**	Floor	0/8	**2/8**	**2/16 (12.5)**
**Farm exterior (total)**	House surrounding	0/8	**2/8**	**2/16 (12.5)**
**Slaughterhouse (total)**	Neck skin	NS	**10/40**	**10/40 (25.0)**
**No. of positive samples/No. of all samples (%)**		**0/40**	**16/80 (20.0)**	**16/120 (13.3)**

Positive samples are shown in bold. NS, not sampled

The environmental samples were collected by swabbing a surface of approximately 50 cm of the area of interest with pre-moistened 3 M Enviro Swabs (3 M, USA). Boot swabs (4136/4137; SodiBox, France) were used to collect floor/litter samples. In each broiler house, two boot swabs were used and pooled. Two water samples (0.5 L each) were collected in sterile bottles from each house in SVs 1 and 3 (all houses) as well as in SVs 4 and 5 in house 3. Two water samples per house were pooled in a single 1-L sample in the laboratory. Fresh fecal samples (*n* = 2) and fur swabs (*n* = 3) were collected from the cats (*n* = 3). Two cats were from farmer A and one cat from farmer B.

### Isolation and confirmation of *Salmonella* Infantis

A total of 656 samples were tested. *Salmonella* was isolated using the standard methods ISO 6579-1:2017 and ISO 6579-1:2017/Amd 1:2020. Samples were diluted 1:9 in buffered peptone water (BPW; Biolife, Italy) for pre-enrichment. In the case of water samples, 2 × 0.5 L of the sample was filtered through a 0.22-µm membrane filter, and the filter was supplemented with 90 mL of BPW. Flies (up to 10 flies) were homogenized in 9 mL of BPW and incubated and further processed as the other samples tested.

*S.* Infantis was confirmed by conventional serotyping using ISO 6579-3:2014 or with *S.* Infantis-specific real-time PCR using previously published primers and probe [22].

### WGS of *Salmonella* Infantis and bioinformatic analysis

A total of 69 *S*. Infantis isolates were typed using WGS, of which 65 isolates were obtained during SVs 1–7 and four isolates were obtained during official sampling in this farm (three isolates from 2021 from farmers A and B and one isolate from 2022 from farmer C) (Table S3). Genomic DNA was extracted using a DNA Blood & Tissue Kit (Qiagen, Hilden, Germany). DNA libraries were prepared with the Illumina TruSeq DNA Nano Library Prep Kit (Illumina, San Diego, CA, USA). Sequencing was performed on the NovaSeq 6000 System using the 2 × 150 bp chemistry (Illumina, San Diego, CA, USA) to a minimum coverage of 150×. Sequencing data were submitted to the NCBI Sequence Read Archive (SRA) database under the BioProject accession number PRJNA1153991. Run accession numbers for the individual isolates are listed in Table S3. Raw reads were assembled with shovill v1.18 using the --trim option and SPAdes v3.13.1 [23] as the underlying assembler with default parameters. Quality of the assemblies was assessed using Quast v5.0.2 [24]; only the genomes with *N*_50_ > 20,000 bp, number of contigs (longer than 1000 bp) less than 500 and total assembly length of ~ 2.8 Mbp were included into further analyses.

Antimicrobial resistance genes were identified using ResFinder v4.1 [25] with default parameters. Multi-locus sequence typing (MLST) was performed using the Sequence query tool implemented in the *Salmonella* spp. PubMLST database [26]. The presence of pESI-like plasmid was defined as the presence of pESI backbone genes *hyp*, *fim* and K88 [14], as determined by BLASTn. The core genome MLST (cgMLST) analysis was performed using chewBBACA v2.8.5 [27]; to maximize the discriminatory power, an ad hoc cgMLST scheme was constructed, and core loci were defined as loci present in 95% genomes under study. A neighbor-joining tree based on cgMLST allele profiles was constructed using Grapetree v1.5 [28]. A threshold of 7 cgMLST allele differences was used for cluster delineation [29]. In silico serotyping was performed using SeqSero2 v1.1.0 [30].

### Statistical analysis

Fisher’s exact test, which was used to compare the differences in the occurrence of *S.* Infantis between different areas of the broiler farm and different SVs, was performed in R statistical software v4.2.1 using the Benjamini-Hochberg correction of the *p*-value.

## Results

### Isolation of *Salmonella* Infantis

The results of the bacteriological examination of samples collected in 2021 and 2022 are shown in Tables 1 and 2, S1 and S2. All *Salmonella* isolates were confirmed as *S.* Infantis by conventional serotyping or real-time PCR. In SV 1, three critical points were observed in the production area: floor/litter (25% of samples positive), ventilation system (fans and air flaps; 24% of samples positive) and nipple cups (50% of samples positive) (Table 1). In all SVs, the floor samples from the anteroom were frequently contaminated (24.4% of samples positive), including the disinfection bath in house 3 (Tables 1 and 2). Equipment surfaces and cleaning tools were positive for *S.* Infantis in 5.9% but were only positive in houses 1–3 (farmer A). *S.* Infantis was also present in the farm exterior of houses 1–6 (farmers A and B; 11.8% of samples positive). All samples from houses 7 and 8 (farmer C) were negative for *S.* Infantis until the last SV 7, except for a floor sample from the anteroom in house 7 (Table 3). The trucks and farmers’ vehicles as well as the farmers themselves (e.g. shoes) were often positive (7.9%) for *S.* Infantis. Sampling in 2021 showed that the C&D procedures were effective between rearing cycles 1 and 2, although the equipment surface in the anteroom of house 3 was positive both before and after C&D. Overall, the occurrence of *S.* Infantis was lower after rearing cycle 2 (5.9%) than after rearing cycle 1 (19.4%) as additional recommendations for C&D were proposed before rearing cycle 2. Prior to the housing of broilers for rearing cycles 3 (year 2021) and 4 (year 2022), all houses were negative for *S.* Infantis. However, the re-introduction of *S.* Infantis occurred in houses 1, 6, 7 and 8 during rearing cycle 4.

**Table 3:** ccurrence of* Salmonella* Infantis in the sampled houses during sampling visits 1–7.

		No. of *S*. Infantis isolates	Sampling visit	Production area	Anteroom	Farm exterior	Slaughterhouse
Farmer A	House 1	4	1		Pos.		
			7			Pos.	Pos.
	House 2	11	1	Pos.	Pos.	Pos.	
			4		Pos.		
	House 3	19	1	Pos.	Pos.	Pos.	Pos.
			3		Pos.		
			4	Pos.	Pos.	Pos.	Pos.
Farmer B	House 4	6	1	Pos.	Pos.	Pos.	
			2	Pos.			
	House 5	1	1				Pos.
	House 6	14	1	Pos.	Pos.		Pos.
			7		Pos.		Pos.
Farmer C	House 7	9	1		Pos.		
			7	Pos.	Pos.	Pos.	Pos.
	House 8	1	7	Pos.			

Pos., positive for *S*. Infantis

**Fig. 2:**
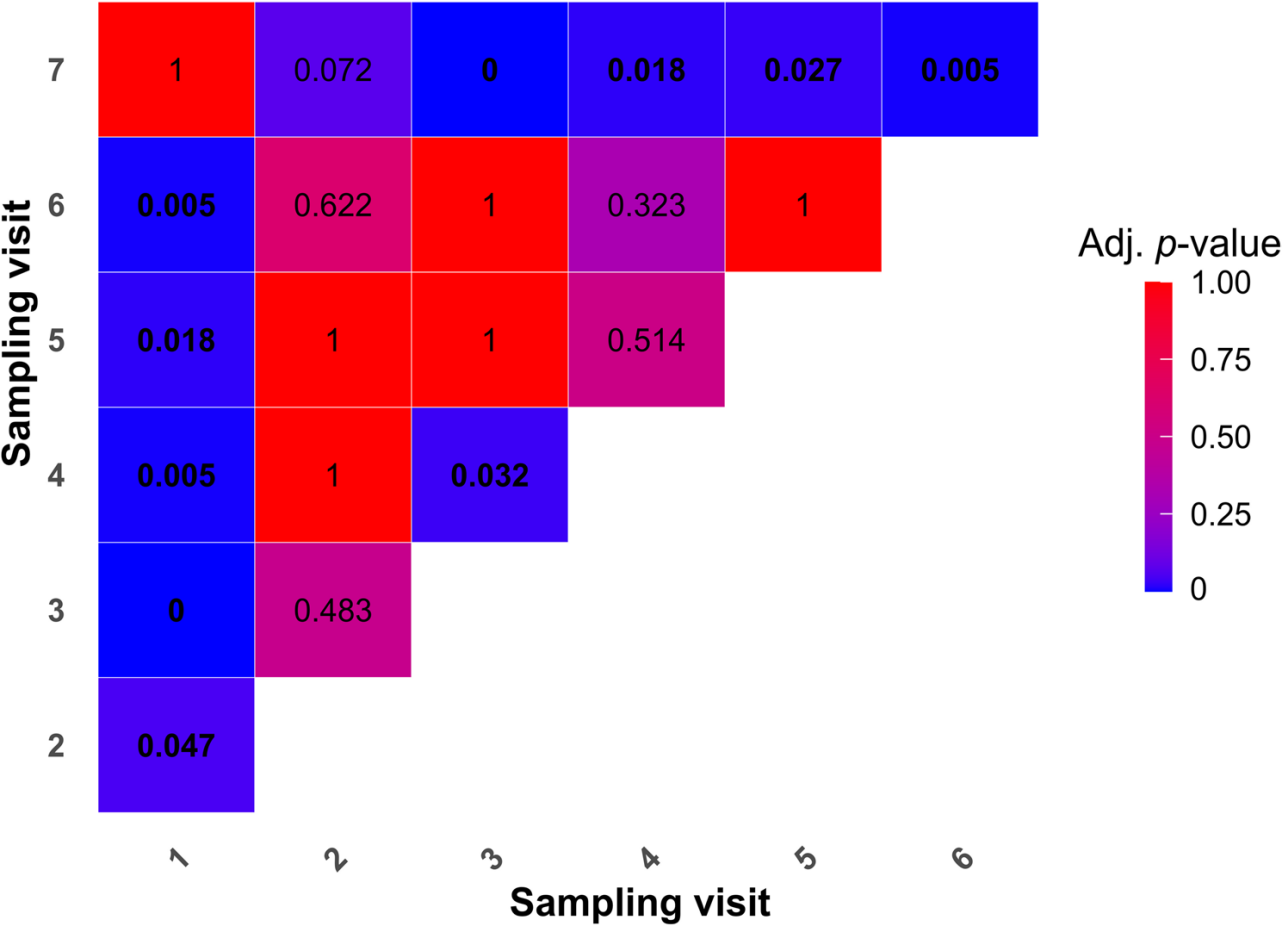
Pairwise comparison of the *Salmonella* Infantis occurrence rate between different sampling visits (Fisher’s exact test). The values in the cells denote the adjusted *p*-values; significant values (*p* < 0.05) are highlighted in bold.

The statistical analysis revealed no significant differences in the four main areas sampled, namely the production area, anteroom, farm exterior and slaughterhouse (Fisher’s exact test, adjusted *p* > 0.1070). When different SVs were compared, the occurrence rate of *S.* Infantis was significantly higher in SV 1 (destocking at the end of rearing cycle 1) and in SV 7 (destocking at the end of rearing cycle 4) than in the other SVs (Fig. 2).

### WGS typing of Salmonella Infantis

The 69 isolates under study were genetically highly homogeneous. All isolates belonged to ST32, harbored pESI-like megaplasmid and were identified as serovar Infantis by in silico serotyping, confirming the results of conventional serotyping or real-time PCR.

**Fig. 3:**
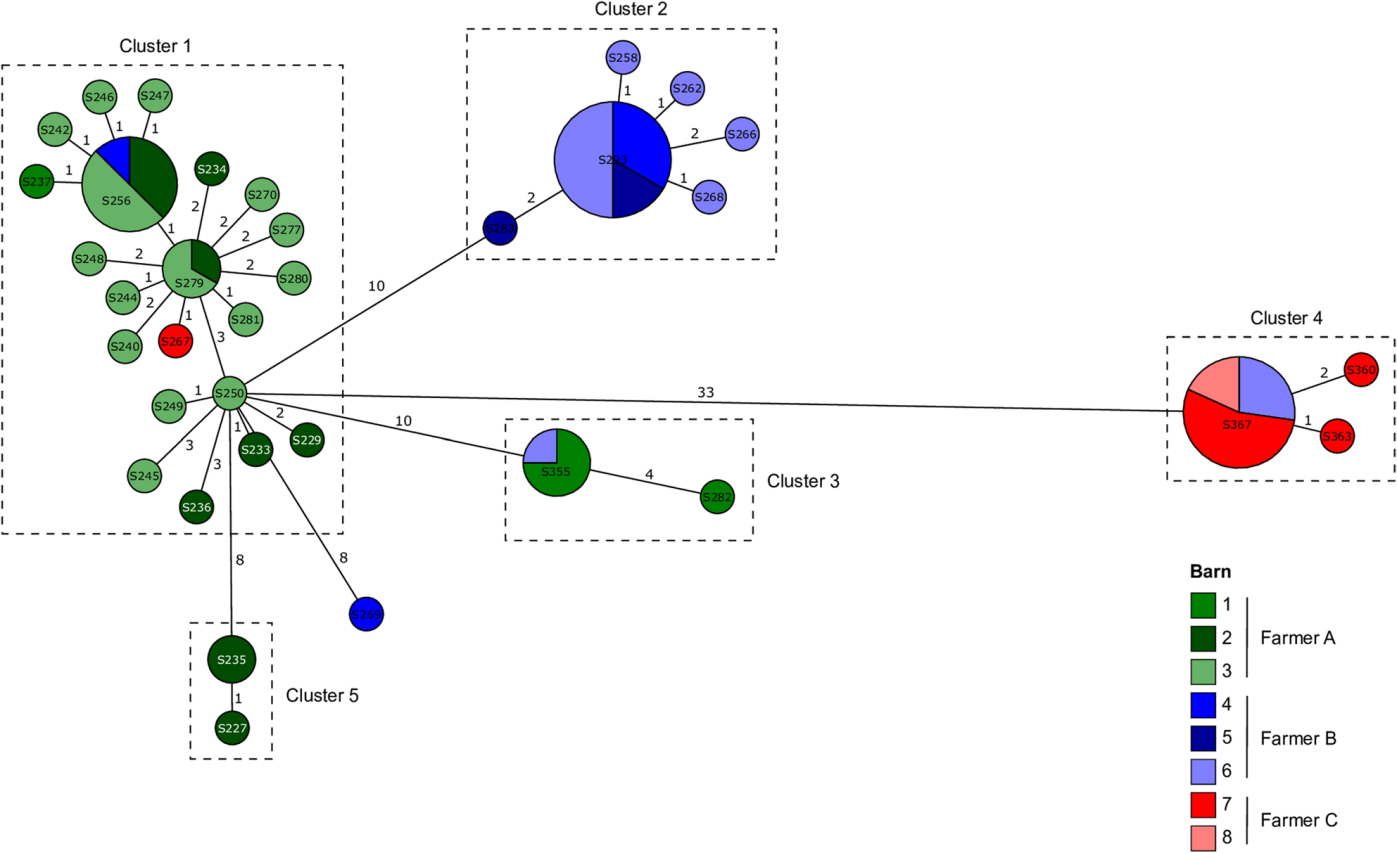
cgMLST tree of the 69 Salmonella Infantis isolates. Clusters of isolates differing in ≤ 7 alleles are denoted.

**Fig. 4:**
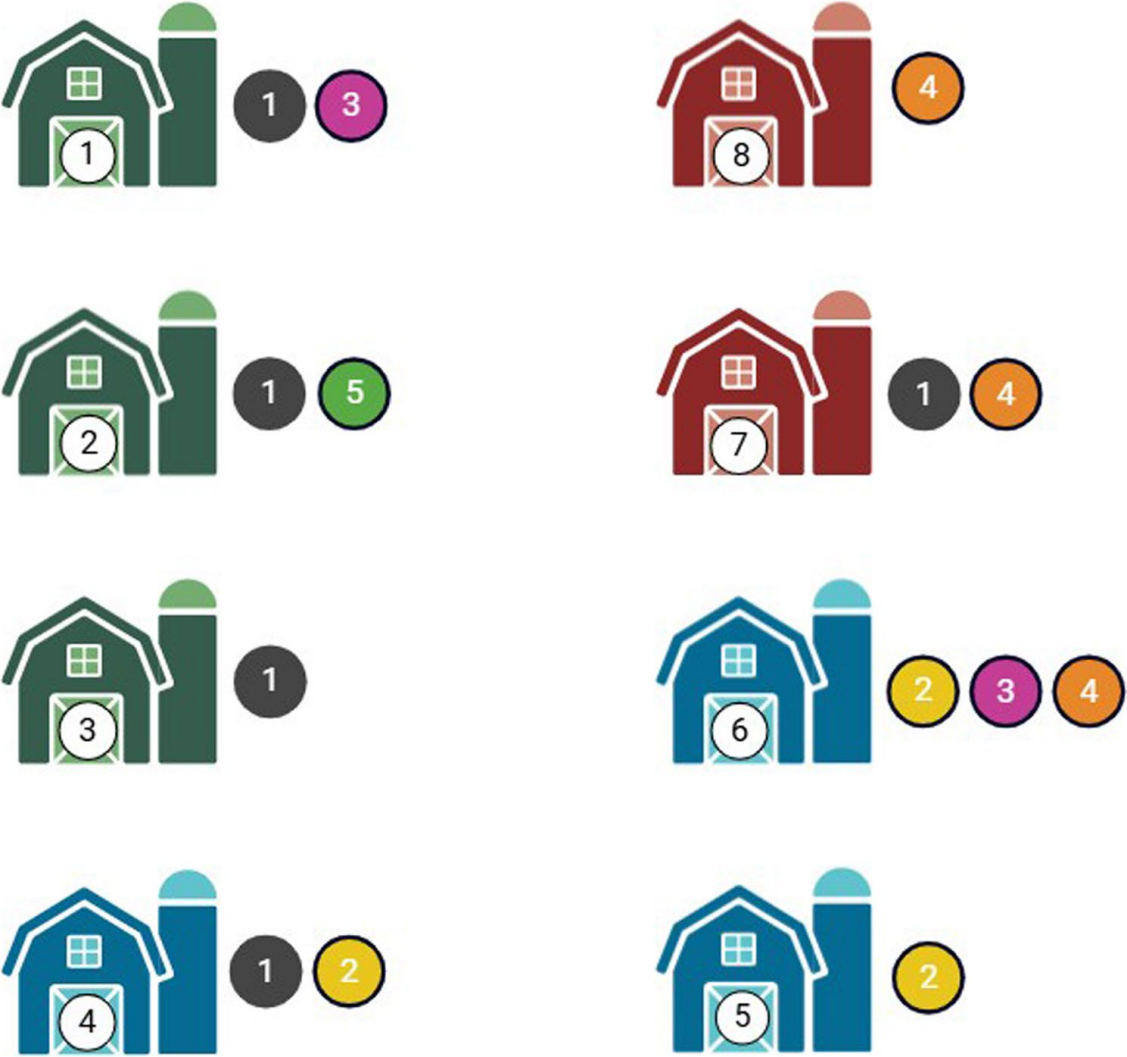
Distribution of the identified clusters of *Salmonella* Infantis isolates across the examined broiler houses. The white node within the house indicates the house number, and the colored nodes next to the houses indicate the arbitrary numbers assigned to the identified *S*. Infantis clusters. Houses of the same color were maintained by the same farmer. The position of the houses reflects their actual arrangement.

The cgMLST analysis revealed the presence of five clusters of clonal isolates comprising 3–30 isolates per cluster. One isolate from house 4 did not fall within the five identified clusters. Clusters 1, 3 and 5 mainly comprised isolates from houses maintained by farmer A, although clusters 1 and 3 also included isolates from houses maintained by multiple farmers. Cluster 2 included isolates from houses maintained by farmer B. Cluster 4 mainly comprised isolates from houses maintained by farmer C, although some isolates were also from the neck skin samples from the slaughterhouse of farmer B (Figs. 3 and 4). All isolates harbored the chromosomally encoded cryptic antimicrobial resistance gene (ARG) *aac(6´)-Iaa*, and all isolates harbored the acquired ARGs *tet*(A), *aadA1* and *sul1* encoding resistance to aminoglycosides (streptomycin), tetracyclines and sulfonamides. In addition, all isolates harbored resistance-associated point mutations in *parC* (T57S), *gyrA* (S83Y) and *nfsA* (nonsense mutation at position 159) conferring resistance to fluoroquinolone and nitrofurantoin, respectively. All isolates from cluster 4 also harbored *bla*_TEM−1 C_ encoding resistance to penicillin. All acquired ARGs were located on pESI-associated contigs and were therefore most likely encoded by pESI. When comparing isolates from different flocks maintained by the same farmer, clusters 1–3 were distributed over different rearing cycles. Of note, cluster 3 spread over two consecutive years, indicating its persistence (or constant re-introduction into the premises). Clusters 4 and 5 were limited to a single rearing cycle (Table S4).

## Discussion and conclusions

This study combined bacteriological examination and WGS typing of the obtained isolates to identify the transmission dynamics of *S*. Infantis in a broiler farm. Several critical points were identified. Various matrices within the production area were contaminated, including the ventilation, feeding and drinking system as well as floor/litter. These are all parts of the production area that are frequently contaminated with *Salmonella* spp. and contribute to the horizontal spread of the pathogen within broiler production houses [5, 31].

The anteroom is considered a buffer zone between the production area and the external environment of the poultry house [5]. Biosecurity compliance in barn anterooms is considered a key component in preventing the introduction and spread of pathogens in poultry farms [32, 33]. A previous study demonstrated that the absence of house-specific anterooms poses a risk for *Salmonella* contamination of the poultry house [34]. In this study, samples from the anteroom were frequently positive for *S.* Infantis, and *S.* Infantis was found in all sample types including the disinfection bath and cleaning tools, resulting in the highest rate of *S.* Infantis-positive samples compared with the farm exterior and the production area. This highlights the importance of regular cleaning of the anteroom and its associated inventory as well as regular (daily) maintenance of the disinfection bath. Since the only sample after C&D between rearing cycles 1 and 2 was positive in the anteroom (equipment surfaces) in house 3, special attention to C&D of the anteroom equipment before the next rearing cycle could also be suggested to reduce cross-contamination with *S.* Infantis between rearing cycles.

In the farm exterior, cats, animal transport vehicles and floor/grass as well as farmers and their vehicles were frequently positive for *S*. Infantis in SV 1. As suggested previously, these can all contribute to the re-introduction of *Salmonella* into the poultry farm and the transmission of *Salmonella* between poultry houses [5, 31, 35, 36].

Neck skin samples from the slaughterhouse were also highly positive for *S*. Infantis, underlining that the slaughterhouse is another critical point for the introduction and transmission of *Salmonella* into the food chain [37]. The isolates from the slaughterhouse belonged to four cgMLST clusters, all of which were also identified at the production level (barn interior, anteroom and barn exterior). This shows that the broilers are already contaminated at the production level and that the same strains found in the broilers also enter the slaughterhouse, as has already been shown previously [38]. However, in SV 7, house 6 had the same strain in the slaughterhouse as the one present in houses 7–8 (both at production and slaughterhouse levels) maintained by a different farmer. Because all the investigated houses used the same slaughterhouse and broilers from these three houses were slaughtered on the same day, cross-contamination at the slaughterhouse level is very likely.

Standard C&D protocols are not fully effective in eliminating *S.* Infantis from poultry farms; therefore, special modifications should be implemented regarding the critical points on farms [5]. The effectiveness of C&D procedures implemented in this study was high since the occurrence rate of *S.* Infantis decreased over time between SVs 1 and 4, and all samples were negative during SVs 5 and 6. Based on the results of SV 1 and a detailed inspection of the farm premises in SV 2, critical points were defined in each house and at the farm level. Veterinarians specialized in biosecurity provided expert guidance to each farmer to improve the effectiveness of C&D measures. This is likely to have contributed to the high effectiveness of C&D observed in the first year of the study. Farmers’ adherence to rigidly following C&D guidelines appears to have declined in the second year of the study, resulting in more positive samples found in SV 7. WGS also showed that a new strain was introduced into houses 7 and 8 (both maintained by farmer C) in 2022, which were negative in 2021.

WGS revealed that all isolates belonged to ST32 and formed five cgMLST clades. In general, the isolates from a single cluster originated from houses maintained by a single farmer. However, three out of five clusters contained some isolates from houses maintained by different farmers, suggesting transmission of *S*. Infantis clones between houses maintained by different farmers. Overall, this suggests that while most transmission events can be explained by the farmers’ activities, transmission between houses maintained by different farmers also occurs. Such transmission can be explained by the fact that the houses were in close proximity to each other and that the immediate farm surroundings were also heavily contaminated with *S*. Infantis. *Salmonella* spp. strains can persist and circulate in broiler farms [39]. For example, *Salmonella* spp. can survive for several months in poultry litter-amended soil from agricultural fields and in fresh water [40–43]. We determined that three out of five clusters spanned multiple rearing cycles, and one cluster spanned over two consecutive years, indicating its persistence in the studied broiler farm or continuous re-introduction into the farm from a common reservoir. The presence of persistent strains also indicates that the implemented C&D strategies were not sufficient to completely eradicate *S*. Infantis from the broiler farm.

All WGS-typed isolates harbored pESI-like megaplasmid, which increases *S.* Infantis fitness under different environmental conditions. The pESI-positive *S.* Infantis strains, which are mainly associated with ST32 or its single-locus variants, show better biofilm formation and different infection dynamics [14, 15]. Our previous study showed that the pESI-positive *S*. Infantis ST32 clone replaced the pESI-negative *S*. Infantis clone in Slovenian broiler flocks in the last decade [13]. This suggests that pESI is an important genetic element that promotes the persistence of pESI-positive *S*. Infantis clones in broiler flocks and makes the eradication of *S*. Infantis in poultry flocks extremely difficult. In addition, this clone is also considered an emerging multidrug-resistant pathogen in humans worldwide, which underlines its clinical importance [44], [45].

In conclusion, this study shows that both the interior and exterior of a broiler farm are frequently contaminated with *S*. Infantis. Several cases of *S.* Infantis transmission between different houses were observed, including houses maintained by different farmers, emphasizing the importance of *S.* Infantis transmission between houses. In addition to the commonly used C&D measures in the production area, additional/improved sanitary protocols should also be implemented in areas other than the production area, e.g. improved rodent and pest control on the farm, improved C&D of animal transport vehicles and the anteroom. In addition, farmers should be continuously trained by biosecurity specialists to strictly follow C&D guidelines, in combination with regular assessments of the C&D measures applied on-farm.

## Supplementary Information

Supplementary Material 1.

## Data Availability

No datasets were generated or analysed during the current study.

## Abbreviations

ARG: Antimicrobial resistance gene
BPW: Buffered peptone water
C&D: Cleaning and disinfection
cgMLST: Core genome multi-locus sequence typing
MLST: Multi-locus sequence typing
pESI: Plasmid of emerging *Salmonella* Infantis
SV: Sampling visit
WGS: Whole-genome sequencing
